# Family income and cardiovascular disease risk in American adults

**DOI:** 10.1038/s41598-023-27474-x

**Published:** 2023-01-06

**Authors:** Abdul Mannan Khan Minhas, Vardhmaan Jain, Monica Li, Robert W. Ariss, Marat Fudim, Erin D. Michos, Salim S. Virani, Laurence Sperling, Anurag Mehta

**Affiliations:** 1 Department of Medicine, Forrest General Hospital, Hattiesburg, MS USA; 2 Emory Clinical Cardiovascular Research Institute, Emory University School of Medicine, Atlanta, GA USA; 3 Department of Medicine, Emory University School of Medicine, Atlanta, GA USA; 4 Department of Medicine, Brigham and Women’s Hospital, Boston, MA USA; 5 Division of Cardiology, Duke University Medical Center, Durham, NC USA; 6 Duke Clinical Research Institute, Durham, NC USA; 7 Division of Cardiology, Johns Hopkins University School of Medicine, Baltimore, MD USA; 8 Michael E. DeBakey Veterans Affair Medical Center and Section of Cardiovascular Research, Department of Medicine, Baylor College of Medicine, Houston, TX USA; 9 VCU Health Pauley Heart Center, Virginia Commonwealth University School of Medicine, 1200 East Broad Street, PO Box 980036, Richmond, VA 23298 USA

**Keywords:** Cardiology, Health care

## Abstract

Socioeconomic status is an overlooked risk factor for cardiovascular disease (CVD). Low family income is a measure of socioeconomic status and may portend greater CVD risk. Therefore, we assessed the association of family income with cardiovascular risk factor and disease burden in American adults. This retrospective analysis included data from participants aged ≥ 20 years from the National Health and Nutrition Examination Survey (NHANES) cycles between 2005 and 2018. Family income to poverty ratio (PIR) was calculated by dividing family (or individual) income by poverty guidelines specific to the survey year and used as a measure of socioeconomic status. The association of PIR with the presence of cardiovascular risk factors and CVD as well as cardiac mortality and all-cause mortality was examined. We included 35,932 unweighted participants corresponding to 207,073,472 weighted, nationally representative participants. Participants with lower PIR were often female and more likely to belong to race/ethnic minorities (non-Hispanic Black, Mexican American, other Hispanic). In addition, they were less likely to be married/living with a partner, to attain college graduation or higher, or to have health insurance. In adjusted analyses, the prevalence odds of diabetes mellitus, hypertension, coronary artery disease (CAD), congestive heart failure (CHF), and stroke largely decreased in a step-wise manner from highest (≥ 5) to lowest PIR (< 1). In adjusted analysis, we also noted a mostly dose-dependent association of PIR with the risk of all-cause and cardiac mortality during a mean 5.7 and 5.8 years of follow up, respectively. Our study demonstrates a largely dose-dependent association of PIR with hypertension, diabetes mellitus, CHF, CAD and stroke prevalence as well as incident all-cause mortality and cardiac mortality in a nationally representative sample of American adults. Public policy efforts should be directed to alleviate these disparities to help improve cardiovascular outcomes in vulnerable groups with low family income.

## Introduction

Cardiovascular disease (CVD) remains a leading cause of morbidity and mortality in the United States (US)^1^. The steady decline in CVD-related mortality has plateaued in the past decade despite remarkable progress in domains of CVD diagnosis, treatment, and prevention^1^. In this context, socioeconomic status (SES) is an often overlooked ‘risk factor’ that exerts a tangible influence on cardiovascular outcomes^2^. Income level, educational attainment, employment status, and neighborhood socioeconomic factors are four measures of SES that have been shown to be consistently associated with CVD in high-income countries^2^. Among these, family income is easily measurable and can be standardized against the federal poverty level to calculate the family income-to-poverty ratio (PIR)^3^.

PIR is a validated measure of income disparity and its relation with cardiovascular risk factors has been thoroughly investigated^3–7^. These studies consistently show that economically disadvantaged individuals with low PIR have a relatively higher burden of cardiovascular risk factors. Additionally, studies evaluating temporal trends in the association of PIR with risk factors have reported worsening income related disparities in risk factor prevalence^5–7^. Considering the association with cardiovascular risk factors, PIR may be associated with prevalent CVD and incident all-cause and cardiac mortality. However, there is a paucity of contemporary studies evaluating the independent association of PIR with prevalent CVD and the incident risk of adverse cardiovascular outcomes in the general population. Thus, in the current study we leveraged a nationally representative database to investigate the association of family income with cardiovascular risk factors, three prevalent CVD phenotypes—coronary artery disease (CAD), congestive heart failure (CHF), and stroke as well as incident all-cause and heart disease mortality in US adults. We utilized the National Health and Nutrition Examination Survey (NHANES) to categorize participants based on their financial status by utilizing PIR. The objectives of this study included investigating socio-demographic characteristics stratified by PIR and association of PIR with hypertension, diabetes mellitus, obesity, dyslipidemia, stroke, CHF, CAD, cardiac mortality, and all-cause mortality. We also assessed trends in cardiovascular risk factors and CVD stratified by PIR from 2005 to 2018.

## Methods

### Data sharing statement

All data used in the current analysis are publicly available through the National Center for Health Statistics and can be accessed at https://wwwn.cdc.gov/nchs/nhanes/default.aspx.

### Data source

NHANES collects nationally representative data of the civilian, non-institutionalized, US population in a 2-year cycle. The NHANES uses a complex, multi-stage, probability sampling design and collects information from approximately 5000 persons per year. Every participant provides informed consent, and the study was conducted in accord with the principles of the Declaration of Helsinki. Institutional review board of the National Center for Health Statistics approves the protocol. The survey consists of a structured home interview, followed by a standardized health examination that includes a physical examination as well as laboratory tests conducted at a mobile examination center. The NHANES study design, operation, and contents have been published previously and are available online^8,9^. We used data for adult participants aged ≥ 20 years from seven NHANES cycles from 2005 to 2018 for the current analysis. Our study used publicly available, deidentified data, hence it was exempt from institutional review board approval.

### Study population and study variables

We included all participants ≥ 20 years of age who did not have missing information on PIR (unweighted n = 35,932). PIR was used as a measure of participant financial status. The Department of Health and Human Services poverty guidelines were used as the poverty measure to calculate this ratio. PIR was calculated by dividing family (or individual) income by the poverty guidelines specific to the survey year. The value was not computed if the respondent only reported income as < $20,000 or ≥ $20,000. Values at or above 5.00 were coded as 5.00 or more because of disclosure concerns. The values were not computed if the income data was missing. We converted the ratio to a categorial variable of 6 levels: PIR < 1 (for the lowest income group), 1–1.9, 2–2.9, 3–3.9, 4–4.9, ≥ 5 (for the highest income group).

We examined several variables routinely collected in NHANES. This included socio-demographic variables (age, gender, health insurance, level of education, marital status, citizenship status). We also examined smoking status and marijuana use of the participants. Cardiovascular and other chronic health conditions were defined by the participants’ response to a series of questions as a part of the standardized questionnaire: CHF: *“Has a doctor or other health professional ever told you that you had congestive heart failure?”*^9^; Stroke: *“Has a doctor or other health professional ever told you that you had a stroke?”*; CAD: *“Has a doctor or other health professional ever told you that you had coronary heart disease?”*, *“Has a doctor or other health professional ever told you that you had angina, also called angina pectoris?”, “Has a doctor or other health professional ever told you that you had a heart attack (also called myocardial infarction).* Measurements of height, weight, and blood pressure were performed following a standardized protocol. Body mass index (BMI) was calculated as weight in kilograms divided by height in meters squared (kg/m2). Obesity was defined as BMI ≥ 30 kg/m2. Hypertension was defined as having systolic blood pressure level ≥ 130 mm Hg and/or diastolic blood pressure level ≥ 80 mm Hg or yes to any of the following question: *“Has a doctor or other health professional ever told you that you had had hypertension, also called high blood pressure”*, *“told on 2 or more different visits that you had hypertension, also called high blood pressure?”*, *“Because of your high blood pressure/hypertension, have you ever been told to take prescribed medicine?”, “Are you now taking prescribed medicine”.* Diabetes mellitus was defined as having a hemoglobin A1c level ≥ 6.5% or serum glucose level ≥ 200 mg/dl or yes to any of the following question *“Other than during pregnancy, have you ever been told by a doctor or health professional that you have diabetes or sugar diabetes?”, “Are you taking insulin now”, “Are you now taking diabetic pills to lower blood sugar”*. Dyslipidemia was defined as total cholesterol level ≥ 240 mg/dl or a participant answering yes to the question *“Have you ever been told by a doctor or other health professional that your blood cholesterol level was high?”*, *“To lower your blood cholesterol, have you ever been told by a doctor or other health professional to take prescribed medicine?”.* Waist circumferences of 102 cm or more for men and 88 cm or more for women were considered high.

### Outcomes

We studied the association of PIR with the presence of cardiovascular risk factors (hypertension, diabetes mellitus, obesity, dyslipidemia), CVD (stroke, CHF, CAD), cardiac mortality, and all-cause mortality at the longest duration of follow-up. We combined the NHANES data with the cause of death from probabilistically linked death certificate records provided by the National Center of Health Statistics from the National Death Index^10^. The public-use linked mortality file for 1999–2014 NHANES includes follow-up time and the underlying cause of death for NHANES adult participants through December 31, 2015. Hence, the mortality analysis was restricted to participants included until 2014^9^. Cardiac deaths were defined as those with *International Classification of Diseases, 10th Revision* (ICD-10) codes I00 to I09, I11, I13, and I20 to I51. As a secondary exploratory analysis, we sought to evaluate the temporal trends in cardiovascular risk factors and CVD by categories of PIR across NHANES cycles from 2005 to 2018.

### Statistical analysis

Descriptive statistics were used to summarize continuous and categorical variables. The weighted mean and standard error were used for continuous variables, and the categorical variables were expressed as unweighted frequencies and weighted percentages. Univariable analyses for between-group comparisons were performed using the Rao-Scott Chi-square test for categorical variables (e.g., sex and citizenship status) and weighted simple linear regression for continuous variables (e.g., age). Weighted logistic regression was performed to estimate unadjusted and adjusted odds ratios (ORs and 95% confidence intervals [CIs]) for the cross-sectional association of PIR and various outcomes including presence of dyslipidemia, diabetes mellitus, obesity, hypertension, CHF, CAD, and stroke. PIR < 1 was used as the reference category. The first logistic regression model was adjusted for age, sex, and race/ethnicity. Model 2 was adjusted for the variables used in Model 1 along with citizenship status, marital status, education status, and insurance status (yes or no). Model 3 was adjusted for all the variables in Model 2 along with diabetes mellitus, hypertension, smoking status, dyslipidemia, and obesity. PIR was also evaluated as a continuous variable. Additionally, restricted cubic splines (RCS) were used to explore potential nonlinear relationships between PIR as a continuous variable and presence of cardiovascular risk factors and CVD. The RCS models used 4 knots with the relationship plotted as odds ratio and 95% CIs for outcomes on the y-axis and PIR on the x-axis. The default knot values (0.31, 1.22, 2.71, 5) were provided as default by STATA based on Harrell's recommended percentiles.

We used univariable Cox proportional-hazards models to assess the association of PIR with all-cause and cardiac mortality. PIR < 1 was used as the reference category. Hazard ratios (HRs) and 95% CIs were estimated. Follow-up began at the time of interview and ended on the date of death or December 31, 2015, whichever came first. We used multivariable Model 1 to adjust for age, sex and race/ethnicity. Model 2 was adjusted for all the variables in Model 1 plus citizenship status, marital status, education status and insurance status (yes or no). Model 3 was adjusted for all the variables in Model 2 plus diabetes mellitus, hypertension, smoking status, dyslipidemia and obesity. Model 4 was adjusted for all the variables in Model 3 plus CAD, stroke and CHF.

Using STATA 16.1 (StataCorp, College Station, TX)^11^, our analyses took into account the NHANES survey design complexity by incorporating sampling weights, primary sampling units, and strata. This allowed us to estimate population proportions, means, and regression coefficients using *svy* commands. Appropriate sampling weights for each analysis were used as designated and described in detail in the NHANES methodology handbook. Standard errors (SEs) were computed using Taylor series linearization. Two-sided *p*-values < 0.05 were considered statistically significant.

## Results

We included 35,932 unweighted participants from the NHANES cycles 2005 to 2018 corresponding to 207,073,472 weighted, nationally representative American adults. The mean age was 47.4 (SE: 0.2) years, 52% of participants were women, 68% were Non-Hispanic White, and 11% were Non-Hispanic Black. The demographic characteristics as well as distribution of cardiovascular risk factors of the study sample stratified by categories of PIR are given in Table 1. Notably, participants with lower PIR were younger (PIR < 1: 41.5 years vs. PIR ≥ 5: 48.6 years), more often female (PIR < 1: 56.7% vs. PIR ≥ 5: 48.5%), and were more likely to belong to race/ethnic minorities [Mexican American (PIR < 1: 17.2% vs. PIR ≥ 5: 2.4%); Other Hispanic (PIR < 1: 10.3% vs. PIR ≥ 5: 2.4%); and Non-Hispanic Black (PIR < 1: 19.3% vs. PIR ≥ 5: 5.5%]. They were less likely to be married/living with a partner (PIR < 1 : 45.4% vs. PIR ≥ 5: 77.1%), to be a college graduate or above (PIR < 1: 7.5% vs. PIR ≥ 5: 57.7%) or to have some form of health insurance (PIR < 1: 62.6% vs. PIR ≥ 5: 96.4%) (Table 1). They were also more likely to be current daily smokers (PIR < 1: 30.1% vs. PIR ≥ 5: 7.9%) or marijuana users (PIR < 1: 21.2% vs. PIR ≥ 5: 9.4%) compared with participants belonging to the higher PIR category. The unadjusted prevalence of cardiovascular risk factors and CVD stratified by PIR categories is shown in Table 2.

**Table 1:** Baseline and Demographic characteristics of participants stratified by PIR. Variables.

	PIR < 1	PIR 1–1.9	PIR 2–2.9	PIR 3–3.9	PIR 4–4.9	PIR ≥ 5	*p* value
Weighted n (%)	29,708,995 (14.35)	42,772,040 (20.66)	31,870,803 (15.39)	27,317,399 (13.19)	21,415,059 (10.34)	53,989,175 (26.07)	
Unweighted n (%)	7703 (21.44)	9674 (26.92)	5497 (15.3)	3983 (11.08)	2798 (7.79)	6277 (17.47)	
Age (mean [S.E])*	41.51 (0.3)	47.79 (0.3)	48.00 (0.3)	47.02 (0.3)	46.82 (0.4)	48.62 (0.2)	< 0.001
**Age groups**							< 0.001
20–39	3210 (50.3)	3245 (40.3)	1880 (39.0)	1336 (36.0)	906 (34.0)	1757 (27.6)	
40–59	2321 (31.4)	2770 (30.5)	1566 (31.6)	1368 (39.0)	1017 (41.430)	2582 (47.7)	
60–79	1806 (15.0)	2781 (22.0)	1579 (23.6)	1022 (21.5)	718 (21.44)	1652 (22.4)	
> = 80	366 (3.4)	878 (7.2)	472 (5.9)	257 (3.8)	157 (3.0)	286 (2.3)	
**Gender**							< 0.001
Men	3418 (43.3)	4,648 (45.6)	2732 (48.9)	1956 (48.3)	1403 (50.0)	3271 (51.5)	
Women	4285 (56.7)	5026 (54.4)	2765 (51.1)	2027 (51.7)	1395 (49.9)	3006 (48.5)	
**Race/ethnicity**							< 0.001
Mexican American	1733 (17.2)	1794 (12.9)	806 (7.9)	439 (5.6)	243 (3.9)	384 (2.4)	
Other hispanic	971 (10.3)	909 (6.8)	519 (5.4)	311 (4.2)	222 (3.7)	350 (2.4)	
Non-hispanic white	2383 (45.0)	4004 (58.2)	2322 (67.8)	1820 (71.7)	1397 (77.5)	3553 (82.4)	
Non-hispanic black	1904 (19.3)	2070 (14.3)	1272 (11.8)	919 (10.3)	569 (8.1)	990 (5.5)	
Other race—including multi-racial	712 (8.2)	897 (7.9)	578 (7.2)	494 (8.3)	367 (6.8)	1000 (7.4)	
**Citizenship status**							< 0.001
Citizen by birth or naturalization	5870 (80.1)	8107 (87.8)	4823 (92.4)	3685 (95.4)	2592 (95.6)	5874 (96.2)	
Not a citizen of the US	1802 (19.9)	1556 (12.2)	669 (7.6)	294 (4.7)	205 (4.4)	400 (3.8)	
**Marital status**							< 0.001
Married/living with partner	3545 (45.4)	5247 (53.9)	3373 (62.4)	2614 (66.9)	1874 (69.3)	4662 (77.1)	
Widowed/divorced/separated	2165 (25.2)	2738 (26.2)	1209 (20.0)	732 (16.5)	478 (15.2)	800 (10.7)	
Never married	1988 (29.4)	1688 (19.9)	910 (17.6)	635 (16.6)	446 (15.5)	811 (12.2)	
**Education level**							< 0.001
Less than 9th grade	1629 (14.8)	1375 (9.5)	422 (4.2)	161 (2.4)	66 (1.1)	60 (0.5)	
9-11th grade (Includes 12th grade with no diploma)	1830 (22.8)	1797 (16.5)	696 (10.1)	357 (7.4)	199 (5.2)	202 (2.8)	
High school graduate/GED or equivalent	1899 (27.4)	2628 (29.4)	1501 (30.0)	904 (23.8)	525 (19.9)	777 (13.0)	
Some college or AA degree	1816 (27.5)	2848 (32.5)	1860 (36.0)	1436 (37.3)	960 (34.2)	1697 (26.1)	
College graduate or above	517 (7.5)	1015 (12.2)	1012 (19.7)	1123 (29.1)	1048 (39.5)	3541 (57.7)	
**Insurance status**							< 0.001
Yes	4866 (62.6)	7015 (71.9)	4497 (82.5)	3517 (88.3)	2576 (93.2)	6015 (96.4)	
No	2821 (37.4)	2651 (28.1)	995 (17.6)	466 (11.7)	221 (6.8)	260 (3.6)	
**Insurance type**							< 0.001
Private	1332 (20.4)	3724 (42.1)	3323 (64.3)	2893 (75.8)	2179 (82.1)	5311 (87.1)	
Medicare	1646 (16.4)	2820 (24.7)	1459 (21.8)	890 (17.6)	548 (15.7)	1033 (13.1)	
Medicaid	2127 (26.3)	1211 (11.5)	296 (4.4)	111 (1.7)	66 (1.5)	50(0.5)	
**BMI**							< 0.001
< 18.5	184 (2.9)	166 (2.0)	67 (1.3)	46 (1.4)	29 (1.1)	63 (0.9)	
18.5–24.9	1973 (28.5)	2357 (27.0)	1382 (27.0)	1096 (29.0)	716 (26.3)	1863 (31.0)	
25–29.9	2242 (29.8)	2971 (31.0)	1728 (32.5)	1229 (31.2)	919 (34.4)	2116 (35.9)	
≥ 30.0	2905 (38.9)	3680 (40.0)	2044 (39.4)	1466 (38.3)	1011 (38.1)	1943 (32.2)	
**Waist circumference**							< 0.001
Normal	2923 (44.2)	3480 (40.5)	2106 (41.4)	1613 (43.0)	1128 (42.2)	2776 (47.2)	
High	3998 (55.8)	5271 (59.5)	2914 (58.6)	2077 (57.0)	1469 (57.9)	3006 (52.8)	
**Smoking status**							< 0.001
Never	3816 (47.8)	4998 (51.4)	3038 (51.6)	2385 (57.5)	1641 (57.2)	3953 (62.3)	
Past	1432 (17.1)	2421 (23.6)	1444 (27.5)	959 (24.4)	748 (26.6)	1687 (27.2)	
Current nondaily smoker	381 (5)	387 (4.1)	206 (3.4)	120 (3.2)	100 (3.8)	163 (2.6)	
Current daily smoker	2064 (30.1)	1861 (21.0)	807 (17.5)	518 (14.9)	308 (12.6)	472 (7.9)	
**Marijuana**							< 0.001
Never	2110 (41.7)	2413 (42.3)	1357 (39.2)	1107 (41.6)	738 (37.4)	1575 (36.3)	
Former	1544 (37.1)	1862 (39.4)	1189 (45.6)	1022 (47.4)	760 (49.5)	1879 (54.3)	
Current	896 (21.2)	838 (18.3)	416 (15.2)	259 (11.0)	203 (13.2)	346 (9.4)	

*Data are presented as unweighted n (weighted percentage) for categorical variables and weighted mean and s.e for continuous variables.

Key: *PIR*: Family poverty to income ratio, *SE*: standard error, *US*: United States, *GED*: general education development, *AA*: associate of arts, *BMI*: body mass index.

**Table 2:** Cardiovascular risk factor and cardiovascular disease prevalence stratified by PIR.

Variables	PIR < 1	PIR 1- 1.9	PIR 2–2.9	PIR 3–3.9	PIR 4–4.9	PIR ≥ 5	Total	*p* value
Hypertension, n (weighted %)	3881 (47.53)	5340 (52.88)	2982 (51.94)	2089 (51.03)	3158 (48.33)	3,158 (49.18)	18,876 (50.3)	0.001
Total participants	7255	9210	5249	3826	2669	5,980	34,189	
Diabetes, n (weighted %)	1388 (13.97)	1,798 (14.76)	922 (13.15)	599 (11.2)	380 (11.1)	721 (8.96)	5,808 (12.04)	< 0.001
Total participants	7703	9673	5497	3983	2789	6,277	35,931	
Dyslipidemia, n (weighted %)	2683 (31.84)	3901 (37.82)	2292 (40.83)	1629 (39.84)	1139 (40.07)	2,758 (44.42)	14,402 (39.65)	< 0.001
Total participants	7565	9533	5409	3921	2768	6,250	35,446	
Stroke, n (weighted %)	371 (4.12)	509 (4.32)	238 (3.58)	122 (2.39)	77 (1.71)	134 (1.5)	1451 (2.92)	< 0.001
Total participants	7686	9664	5488	3978	2795	6,274	35,885	
Obesity, n (weighted %)	2905 (38.93)	3680 (40.03)	2044 (39.44)	1466 (38.34)	1011 (38.14)	1943 (32.22)	13,049 (37.33)	< 0.001
Total participants	7304	9174	5221	3837	2675	5985	34,196	
CHF, n (weighted %)	310 (3.29)	451 (3.96)	194 (2.91)	115 (2.06)	56 (1.55)	88 (0.88)	1214 (2.4)	< 0.001
Total participants	7671	9638	5486	3977	2793	6,272	35,837	
CAD, n (weighted %)	635 (6.78)	900 (7.85)	407 (6.67)	241 (5.06)	148 (4.22)	314 (4.2)	2645 (5.82)	< 0.001
Total participants	7697	9673	5496	3981	2798	6,277	35,922	

*Data are presented as unweighted n (weighted percentage).

Key: *PIR*: Family poverty to income ratio, *CAD*: Coronary artery disease, *CHF*: Congestive heart failure.

### Association of income with prevalence of hypertension

On multivariable analysis adjusting for age, race/ethnicity and sex (model 1), compared with individuals with PIR < 1, prevalence odds of hypertension were comparable in participants with PIR 1–1.9 (aOR, 95% CI 0.95 (0.87–1.04), *p* = 0.238); and lower in participants with PIR 2–2.9: (0.87 (0.78–0.97), *p* = 0.016); PIR 3–3.9 (0.85 (0.75–0.95), *p* = 0.007); PIR 4–4.9: (0.76 (0.66–0.87), *p* < 0.001), and PIR ≥ 5: (0.69 (0.62–0.77), *p* < 0.001) (Table 3). When used as a continuous variable, increasing PIR was associated with lower odds of hypertension (0.92 (0.90–0.94), *p* < 0.001) [Supplementary Table 2].

**Table 3:** Odds ratios (95% confidence intervals) for the prevalence of cardiovascular comorbidities stratified by PIR (Reference category PIR < 1).

Variables		OR (95% confidence interval)	*p* value	OR (95% confidence interval)	*p* value	OR (95% Confidence Interval)	*p* value	OR (95% confidence interval)	*p* value
		Unadjusted		Adjusted model 1*		Adjusted model 2#		Adjusted model 3$	
**CHF**	PIR 1–1.9	1.21 (0.99–1.48)	0.063	0.78 (0.64–0.95)	0.015	0.78 (0.64–0.97)	0.023	0.83 (0.71–0.98)	0.048
	PIR 2–2.9	0.88 (0.66–1.18)	0.391	0.57 (0.42–0.77)	< 0.001	0.59 (0.42–0.83)	0.002	0.73 (0.60–0.89)	0.011
	PIR 3–3.9	0.62 (0.46–0.83)	0.002	0.43 (0.32–0.58)	< 0.001	0.46 (0.33–0.64)	< 0.001	0.93 (0.75–1.15)	<0.001
	PIR 4–4.9	0.46 (0.31–0.70)	< 0.001	0.35 (0.23–0.53)	< 0.001	0.38 (0.25–0.59)	< 0.001	0.83 (0.67–1.02)	<0.001
	PIR ≥ 5	0.26 (0.19–0.36)	< 0.001	0.19 (0.13–0.26)	< 0.001	0.22 (0.15–0.33)	< 0.001	1.00 (0.84–1.19)	<0.001
**CAD**	PIR 1–1.9	1.17 (0.99–1.38)	0.063	0.70 (0.59–0.84)	< 0.001	0.69 (0.58–0.83)	< 0.001	0.80 (0.64–1.00)	0.001
	PIR 2–2.9	0.98 (0.82–1.18)	0.841	0.57 (0.46–0.70)	< 0.001	0.58 (0.46–0.72)	< 0.001	0.64 (0.45–0.90)	<0.001
	PIR 3–3.9	0.73 (0.59–0.91)	0.006	0.45 (0.35–0.57)	< 0.001	0.46 (0.36–0.59)	< 0.001	0.50 (0.35–0.71)	< 0.001
	PIR 4–4.9	0.61 (0.47–0.78)	< 0.001	0.39 (0.30–0.51)	< 0.001	0.42 (0.32–0.55)	< 0.001	0.39 (0.25–0.61)	< 0.001
	PIR ≥ 5	0.60 (0.49–0.74)	< 0.001	0.37 (0.30–0.45)	< 0.001	0.41 (0.32–0.52)	< 0.001	0.26 (0.17–0.39)	< 0.001
**Stroke**	PIR 1–1.9	1.05 (0.88–1.26)	0.588	0.7 (0.57–0.85)	0.001	0.69 (0.56–0.84)	< 0.001	0.73 (0.61–0.88)	0.009
	PIR 2–2.9	0.86 (0.68–1.10)	0.231	0.59 (0.45–0.77)	< 0.001	0.59 (0.44–0.78)	< 0.001	0.63 (0.51–0.78)	0.001
	PIR 3–3.9	0.57 (0.42–0.77)	< 0.001	0.42 (0.31–0.57)	< 0.001	0.43 (0.31–0.59)	< 0.001	0.51 (0.40–0.65)	< 0.001
	PIR 4–4.9	0.40 (0.29–0.56)	< 0.001	0.32 (0.23–0.45)	< 0.001	0.33 (0.23–0.46)	< 0.001	0.45 (0.33–0.61)	< 0.001
	PIR ≥ 5	0.35 (0.27–0.46)	< 0.001	0.27 (0.20–0.36)	< 0.001	0.29 (0.21–0.40)	< 0.001	0.50 (0.39–0.63)	< 0.001
**Diabetes**	PIR 1–1.9	1.07 (0.95–1.20)	0.293	0.86 (0.77–0.97)	0.013	0.86 (0.76–0.96)	0.009	0.86 (0.76–0.98)	0.020
	PIR 2–2.9	0.93 (0.80–1.09)	0.366	0.79 (0.67–0.92)	0.003	0.78 (0.67–0.92)	0.003	0.80 (0.67–0.96)	0.015
	PIR 3–3.9	0.78 (0.68–0.89)	< 0.001	0.70 (0.61–0.80)	< 0.001	0.71 (0.61–0.82)	< 0.001	0.71 (0.60–0.84)	< 0.001
	PIR 4–4.9	0.77 (0.63–0.93)	0.008	0.75 (0.62–0.91)	0.004	0.78 (0.64–0.95)	0.012	0.76 (0.62–0.93)	0.008
	PIR ≥ 5	0.61 (0.52–0.70)	< 0.001	0.57 (0.49–0.65)	< 0.001	0.62 (0.53–0.73)	< 0.001	0.62 (0.53–0.73)	< 0.001
**Hypertension**	PIR 1–1.9	1.24 (1.13–1.36)	< 0.001	0.95 (0.87–1.04)	0.238	0.94 (0.86–1.03)	0.163	0.95 (0.86–1.04)	0.239
	PIR 2–2.9	1.19 (1.07–1.32)	0.001	0.87 (0.78–0.97)	0.016	0.87 (0.78–0.97)	0.013	0.88 (0.79–0.98)	0.021
	PIR 3–3.9	1.15 (1.03–1.28)	0.013	0.85 (0.75–0.95)	0.007	0.87 (0.76–0.99)	0.031	0.90 (0.79–1.04)	0.144
	PIR 4–4.9	1.03 (0.91–1.17)	0.607	0.76 (0.66–0.87)	< 0.001	0.81 (0.71–0.92)	0.002	0.83 (0.72–0.95)	0.007
	PIR ≥ 5	1.07 (0.96–1.19)	0.212	0.69 (0.62–0.77)	< 0.001	0.80 (0.71–0.90)	< 0.001	0.86 (0.77–0.98)	0.020
**Obesity**	PIR 1–1.9	1.05 (0.96–1.14)	0.28	1.06 (0.97–1.15)	0.203	1.01 (0.93–1.10?	0.85	1.01 (0.92–1.11)	0.805
	PIR 2–2.9	1.02 (0.92–1.13)	0.673	1.06 (0.96–1.17)	0.252	0.98 (0.89–1.09)	0.737	0.98 (0.88–1.10)	0.738
	PIR 3–3.9	0.98 (0.88–1.09)	0.649	1.04 (0.93–1.15)	0.499	0.98 (0.87–1.09)	0.662	0.99 (0.88–1.12)	0.884
	PIR 4–4.9	0.97 (0.84–1.11)	0.635	1.04 (0.91–1.20)	0.57	1.01 (0.87–1.17)	0.889	1.03 (0.90–1.19)	0.639
	PIR ≥ 5	0.75 (0.67–0.83)	< 0.001	0.81 (0.73–0.90)	< 0.001	0.84 (0.75–0.94)	0.002	0.86 (0.77–0.96)	0.007
**Dyslipidemia**	PIR 1–1.9	1.30 (1.20–1.42)	< 0.001	1.00 (0.93–1.08)	0.903	0.97 (0.90–1.05)	0.481	1.00 (0.92–1.09)	0.917
	PIR 2–2.9	1.48 (1.32–1.66)	< 0.001	1.15 (1.03–1.28)	0.015	1.07 (0.96–1.20)	0.229	1.13 (1.00–1.28)	0.053
	PIR 3–3.9	1.42 (1.26–1.59)	< 0.001	1.12 (0.99–1.26)	0.061	1.03 (0.92–1.16)	0.58	1.10 (0.97–1.25)	0.137
	PIR 4–4.9	1.43 (1.24–1.65)	< 0.001	1.15 (1.01–1.31)	0.038	1.06 (0.92–1.21)	0.452	1.14 (0.98–1.31)	0.082
	PIR ≥ 5	1.71 (1.54–1.90)	< 0.001	1.27 (1.15–1.41)	0.001	1.17 (1.04–1.31)	0.007	1.31 (1.17–1.46)	< 0.001

* Models are adjusted for age, sex and race/ethnicity.

^#^ Models are adjusted for age, sex, race/ethnicity, citizen status, marital status, education status and insurance (yes or no, not the types).

^$^ Models are adjusted for age, sex, race/ethnicity, citizen status, marital status, education status and insurance (yes or no, not the types), diabetes mellitus, hypertension, smoker, obesity, dyslipidemia. In model $, we excluded the outcomes of particular analysis for diabetes mellitus, hypertension, obesity and dyslipidemia as covariate in the model.

Key: PIR: Family poverty to income ratio, CAD: Coronary artery disease, CHF: Congestive heart failure.

### Association of income with prevalence of diabetes mellitus

On multivariable analysis adjusting for age, race/ethnicity and sex (model 1), compared with individuals with PIR < 1, prevalence odds of diabetes were lower in participants with PIR 1–1.9: (aOR, 95% CI 0.86 (0.77–0.97), *p* = 0.013); PIR 2–2.9: (0.79 (0.67–0.92), *p* = 0.003); PIR 3–3.9: (0.70 (0.61–0.80), *p* < 0.001); PIR 4–4.9: (0.75 (0.62–0.91), *p* = 0.004), and PIR ≥ 5: (0.57 (0.49–0.65), *p* < 0.001) (Table 3). When used as a continuous variable, increasing PIR was associated with lower odds of diabetes mellitus (0.90 (0.88–0.93), *p* < 0.001) [Supplementary Table 2].

### Association of Income with Prevalence of dyslipidemia:

On multivariable analysis adjusting for age, race/ethnicity and sex, compared with individuals with PIR < 1, prevalence odds of dyslipidemia were comparable in participants with PIR 1–1.9: (aOR, 95% CI 1.00 (0.93–1.08), *p* = 0.903); higher in participants with PIR 2–2.9: (1.15 (1.03–1.28), *p* = 0.015); comparable in participants with PIR 3–3.9: (1.12 (0.99–1.26), *p* = 0.061); and higher in participants with PIR 4–4.9: (1.15 (1.01–1.31), *p* = 0.038), and PIR ≥ 5: (1.27 (1.15–1.41), *p* 0.001) (Table 3). When used as a continuous variable, increasing PIR was associated with higher odds of dyslipidemia (1.05 (1.03–1.07), *p* < 0.001) [Supplementary Table 2].

### Association of income with prevalence of obesity

On multivariable analysis adjusting for age, race/ethnicity and sex, compared with individuals with PIR < 1, prevalence odds of obesity were comparable in participants with PIR 1–1.9: (aOR, 95% CI 1.06 (0.97–1.15), *p* = 0.203); PIR 2–2.9: (1.06 (0.96–1.17), *p* = 0.252); PIR 3–3.9: (1.04 (0.93–1.15), *p* = 0.499); PIR 4–4.9: (1.04 (0.91–1.20), *p* = 0.57), but lower in participants with PIR ≥ 5: (0.81 (0.73–0.90), *p* < 0.001) (Table 3). When used as a continuous variable, increasing PIR was associated with lower odds of obesity (0.96 (0.94–0.98), *p* < 0.001) [Supplementary Table 2].

### Association of income with prevalence of stroke

On multivariable analysis adjusting for age, race/ethnicity and sex, compared with individuals with PIR < 1, prevalence odds of stroke were lower in participants with PIR 1–1.9: (0.7 (0.57–0.85), *p* = 0.001); PIR 2–2.9: (0.59 (0.45–0.77), *p* < 0.001); PIR 3–3.9: (0.42 (0.31–0.57), *p* < 0.001); PIR 4–4.9: (0.32 (0.23–0.45), *p* < 0.001) and PIR ≥ 5: (0.27 (0.20–0.36), *p* < 0.001) (Table 3). When used as a continuous variable, increasing PIR was associated with lower odds of stroke (0.75 (0.71–0.80), *p* < 0.001) [Supplementary Table 2].

### Association of income with prevalence of CAD

On multivariable analysis adjusting for age, race/ethnicity and sex, compared with individuals with PIR < 1, prevalence odds of CAD were lower in participants with PIR 1–1.9: (aOR, 95% CI 0.70 (0.59–0.84), *p* < 0.001); PIR 2–2.9: (0.57 (0.46–0.70), *p* < 0.001); PIR 3–3.9: (0.45 (0.35–0.57), *p* < 0.001); PIR 4–4.9: (0.39 (0.30–0.51), *p* < 0.001) and PIR ≥ 5: (0.37 (0.30–0.45), *p* < 0.001) (Table 3). When used as a continuous variable, increasing PIR was associated with lower odds of CAD (0.81 (0.77–0.84), *p* < 0.001) [Supplementary Table 2].

### Association of income with prevalence of CHF

On multivariable analysis adjusting for age, race/ethnicity and sex, compared with individuals with PIR < 1, prevalence odds of CHF were lower in participants with PIR 1–1.9: (aOR, 95% CI 0.78 (0.64–0.95), *p* = 0.015); PIR 2–2.9: (0.57 (0.42–0.77), *p* < 0.001); PIR 3–3.9: (0.43 (0.32–0.58), *p* < 0.001); PIR 4–4.9: (0.35 (0.23–0.53), *p* < 0.001) and PIR ≥ 5: (0.19 (0.13–0.26), *p* < 0.001) (Table 3). When used as a continuous variable, increasing PIR was associated with lower odds of CHF (0.71 (0.66–0.75), *p* < 0.001) [Supplementary Table 2].

### Association of income with all-cause and cardiac mortality

On multivariable analysis adjusting for age, race/ethnicity, and sex, compared with individuals with PIR < 1, all-cause mortality was lower in the participants with PIR 1–1.9: (aHR, 95% CI 0.73 (0.63–0.84), *p* < 0.001); PIR 2–2.9: (0.59 (0.50–0.70), *p* < 0.001); PIR 3–3.9: (0.43 (0.35–0.52), *p* < 0.001); PIR 4–4.9: (0.38 (0.30–0.50), *p* < 0.001) and PIR ≥ 5: (0.26 (0.21–0.32), *p* < 0.001) (Table 4) during a mean 5.7 years of follow up. Number of all-cause deaths and follow-up time stratified by PIR categories are shown in Supplementary Table 4. Kaplan–Meier curves for all-cause mortality stratified by PIR Categories are shown in Supplementary Fig. 1.

**Table 4:** Hazard ratios (95% confidence intervals) for the risk of all-cause mortality stratified by PIR Categories.

Variables	HR (95% confidence interval)	*p* value	HR (95% confidence interval)	*p* value	HR (95% confidence interval)	*p* value	HR (95% Confidence interval)	*p* value	HR (95% confidence interval)	*p* value
	Unadjusted		Adjusted Model 1*		adjusted Model 2#		adjusted model 3$		adjusted Model 4	
PIR 1- 1.9	1.26 (1.09–1.44)	0.001	0.73 (0.63–0.84)	< 0.001	0.79 (0.69–0.91)	0.001	0.86 (0.73–1.00)	0.053	0.89 (0.76–1.05)	0.164
PIR 2–2.9	0.98 (0.83–1.16)	0.836	0.59 (0.50–0.70)	< 0.001	0.71 (0.59–0.85)	< 0.001	0.79 (0.65–0.96)	0.02	0.84 (0.69–1.02)	0.082
PIR 3–3.9	0.61 (0.50–0.74)	< 0.001	0.43 (0.35–0.52)	< 0.001	0.53 (0.43–0.66)	< 0.001	0.62 (0.49–0.78)	< 0.001	0.67 (0.53–0.84)	0.001
PIR 4–4.9	0.48 (0.37–0.62)	< 0.001	0.38 (0.30–0.50)	< 0.001	0.49 (0.37–0.64)	< 0.001	0.56 (0.42–0.76)	< 0.001	0.62 (0.46–0.84)	0.002
PIR ≥ 5	0.34 (0.27–0.42)	< 0.001	0.26 (0.21–0.32)	< 0.001	0.36 (0.28–0.46)	< 0.001	0.43 (0.33–0.56)	< 0.001	0.48 (0.37–0.62)	< 0.001

*Models are adjusted for age, sex and race/ethnicity.

^#^Models are adjusted for age, sex, race/ethnicity, citizen status, marital status, education status and insurance (yes or no, not the types).

^$^Models are adjusted for age, sex, race/ethnicity, citizen status, marital status, education status and insurance (yes or no, not the types), diabetes mellitus, hypertension, smoker, obesity, dyslipidemia.

^¶^Models are adjusted for age, sex, race/ethnicity, citizen status, marital status, education status and insurance (yes or no, not the types), diabetes mellitus, hypertension, smoker, obesity, dyslipidemia, CAD, CHF and stroke.

On multivariable analysis adjusting for age, race/ethnicity and sex, compared with individuals with PIR < 1, cardiac mortality was lower in the participants with PIR 1–1.9 (0.65 (0.47–0.90), *p* = 0.009); PIR 2–2.9: (0.47 (0.32–0.70), *p* < 0.001); PIR 3–3.9: (0.24 (0.14–0.40), *p* < 0.001); PIR 4–4.9: (0.31 (0.15–0.57), *p* < 0.001) and PIR ≥ 5: (0.16 (0.09–0.27), *p* < 0.001) (Table 5) during a mean 5.8 years of follow up. Number of cardiac deaths and follow-up time stratified by PIR categories are shown in Supplementary Table 3. Kaplan–Meier curves for cardiac mortality stratified by PIR Categories are shown in Supplementary Fig. 2.

**Table 5:** Hazard ratios (95% confidence intervals) for the risk of cardiac mortality stratified by PIR Categories.

Variables	HR (95% confidence interval)	*p* value	HR (95% confidence interval)	*p* value	HR (95% confidence interval)	*p* value	HR (95% confidence interval)	*p* value	HR (95% confidence interval)	*p* value
	Unadjusted		Adjusted model 1*		Adjusted model 2#		Adjusted model 3$		Adjusted model 4	
PIR 1- 1.9	1.26 (0.92–1.73)	0.156	0.65 (0.47–0.90)	0.009	0.73 (0.53–1.01)	0.06	0.79 (0.55–1.14)	0.213	0.87 (0.60–1.26)	0.471
PIR 2–2.9	0.92 (0.63–1.35)	0.674	0.47 (0.32–0.70)	< 0.001	0.61 (0.40–0.92)	0.019	0.73 (0.46–1.15)	0.176	0.88 (0.55–1.39)	0.574
PIR 3–3.9	0.39 (0.23–0.64)	< 0.001	0.24 (0.14–0.40)	< 0.001	0.32 (0.19–0.55)	< 0.001	0.36 (0.21–0.64)	0.001	0.44 (0.24–0.79)	0.006
PIR 4–4.9	0.43 (0.24–0.79)	0.006	0.31 (0.17–0.57)	< 0.001	0.42 (0.23–0.78)	0.006	0.56 (0.29–1.06)	0.077	0.67 (0.35–1.26)	0.215
PIR ≥ 5	0.23 (0.14–0.39)	< 0.001	0.16 (0.09–0.27)	< 0.001	0.25 (0.14–0.45)	< 0.001	0.34 (0.18–0.63)	0.001	0.42 (0.23–0.77)	0.006

*Models are adjusted for age, sex and race/ethnicity.

^#^Models are adjusted for age, sex, race/ethnicity, citizen status, marital status, education status and insurance (yes or no, not the types).

^$^Models are adjusted for age, sex, race/ethnicity, citizen status, marital status, education status and insurance (yes or no, not the types), diabetes mellitus, hypertension, smoker, obesity, dyslipidemia.

^¶^Models are adjusted for age, sex, race/ethnicity, citizen status, marital status, education status and insurance (yes or no, not the types), diabetes mellitus, hypertension, smoker, obesity, dyslipidemia, CAD, CHF and stroke.

**Figure 1:**
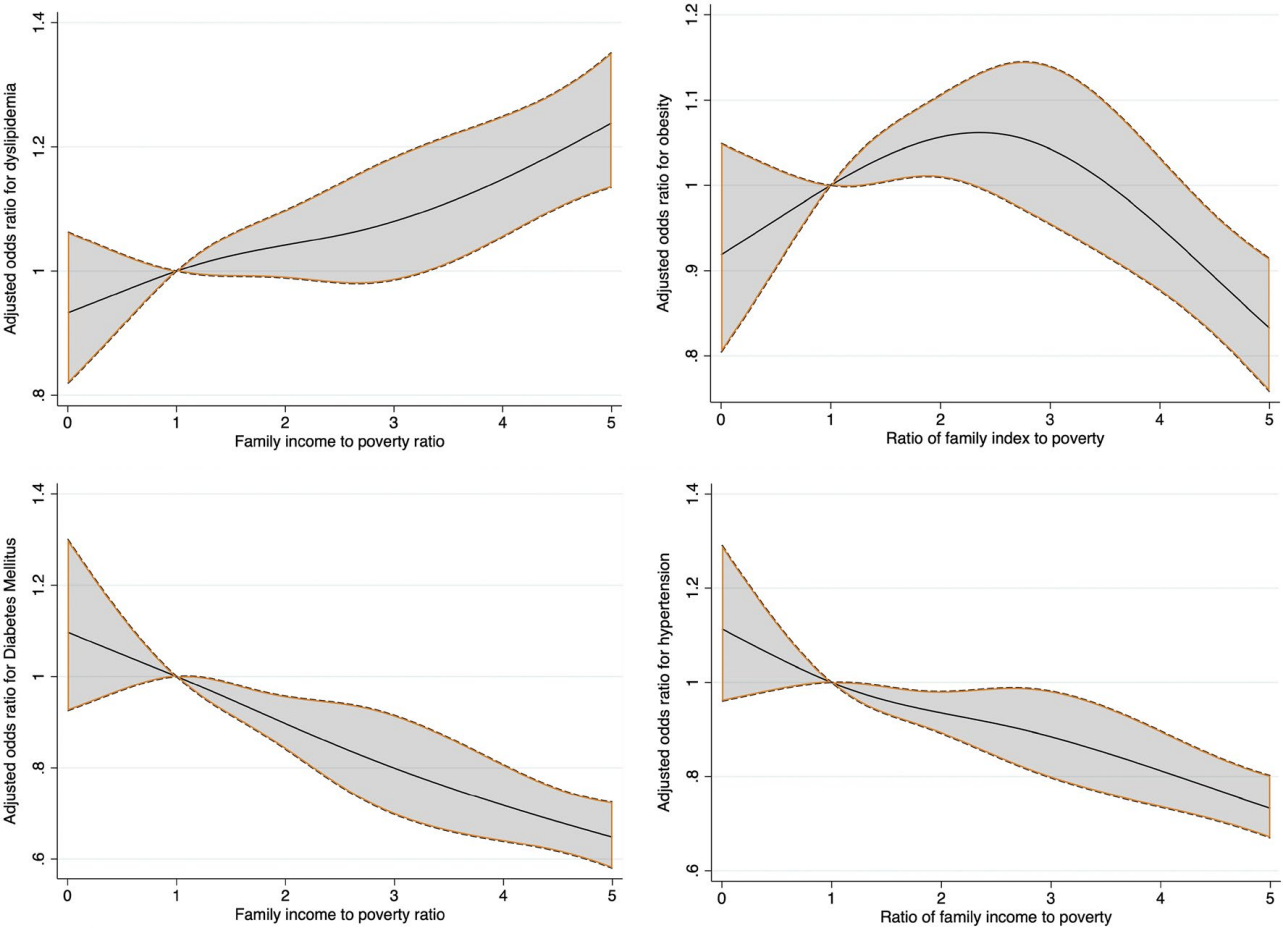
Restricted cubic splines showing relationship between PIR and presence of cardiovascular risk factors.

**Figure 2:**
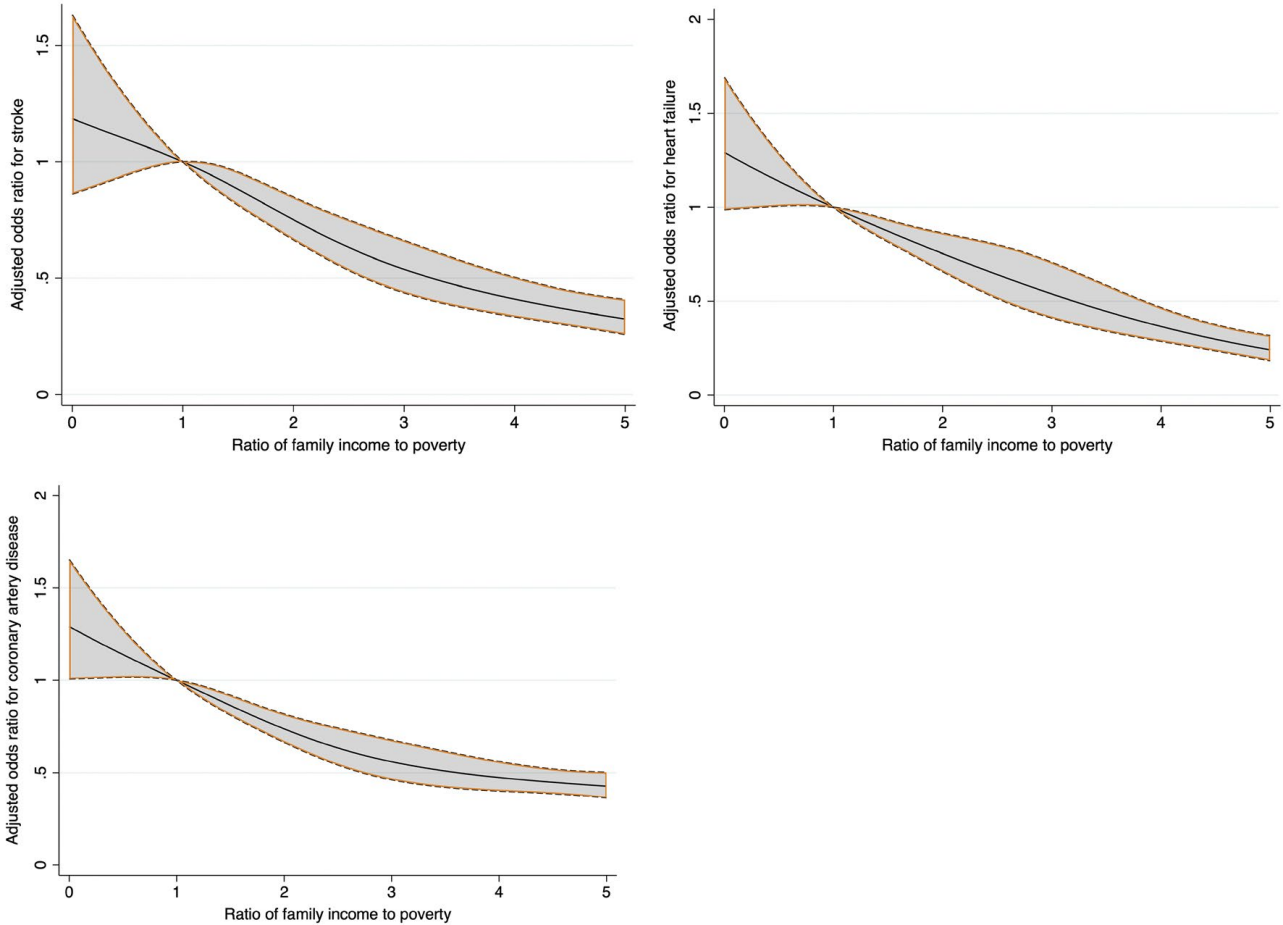
Restricted cubic splines showing relationship between PIR and presence of cardiovascular diseases.

Temporal trends in prevalence of cardiovascular risk factors and CVD stratified by PIR categories are shown in Supplementary Table 1. RCS showing relationship between PIR and cardiovascular risk factors and CVD are shown in Figs. 1 and 2.

## Discussion

In the nationally representative data from NHANES, we report several important findings regarding the association of income disparity and CVD in US adults. First, the lower family income subgroup had a higher proportion of women, race/ethnic minorities, and uninsured individuals. In addition, the lower family income subgroup had higher rates of current smoking, marijuana use, and had lower educational attainment compared with the high family income subgroup. Second, lower income was independently associated with higher odds of diabetes mellitus, hypertension, CAD, CHF, and stroke. Third, we observed that lower income was also independently associated with an increased risk of all-cause and cardiac mortality during follow-up.

Income is one of the major social determinants of health. Income disparity has been studied as a component of SES among NHANES participants in two recent studies. Zhang and colleagues studied NHANES III participants recruited in 1988–1994 and created a cumulative social risk score comprising of PIR < 1, minority race, education < 12th grade, and living single^12^. They observed that a higher cumulative social risk score was independently associated with 19% to 52% higher risk of CVD death^12^. Zhang et al. subsequently studied NHANES participants recruited in 1988–1994 (NHANES III) and 1999–2014 (continuous cycles)^13^. They quantified SES using a latent class analysis comprising of PIR, occupation or employment status, education level, and health insurance^13^. They observed that low SES was associated with more than two-fold higher risk of all-cause and CVD-related mortality in the study population^13^.

We observed a consistent dose-dependent association of PIR with prevalent CAD, CHF, and stroke**.** This relationship was independent of multiple relevant confounders including age, sex, race/ethnicity, cardiovascular risk factors, and importantly four other SES markers – educational attainment, marital status, citizenship status, and health insurance. We noted a similar association of PIR with hypertension and diabetes mellitus. Increased CVD prevalence in the lower income population is likely multifactorial. Social factors including food insecurity, lifestyle factors, lack of appropriate housing/transportation, lack of education/health literacy, inability to afford medications, decreased access to preventative health care, increased prevalence/poorer control of traditional risk factors have been implicated in causing poor health outcomes in low socioeconomic groups^14–17^. Higher CVD prevalence in lower income strata may also attributed to psychosocial stressors and coping behaviors such as drug or alcohol abuse.

Our study corroborates the findings of prior studies which have also documented the relationship between lower SES and cardiovascular morbidity and mortality^18–23^. In a recent seminal study, He et al. recently reported persistent income related disparities among participants of NHANES^7^. They estimated 10-year atherosclerotic CVD risk was significantly higher in the low-income group (PIR < 1)^7^. Abdalla et al. studied participants from nine NHANES cycles between 2009 and 2016, and stratified the study population into two groups using PIR cut-off of 5^24^. They studied the association of income disparity with prevalent CVD and observed that individuals with PIR ≥ 5 had lower prevalence of CVD as compared with those with PIR < 5^24^. This income related disparity remained consistent over time. In contrast to this study, we have studied PIR as a continuous as well as multi-level categorical variable in our analysis and have shown dose-dependent relationship of poverty with CVD prevalence.

We also extend the association of income and CVD to a consistent dose-dependent association between PIR and mortality risk. Kucharska-Newton et al. studied participants of the Atherosclerosis Risk in Communities (ARIC) study recruited in 1987–1989 and observed that low income (< $15,999) was associated with increased risk of sudden and non-sudden cardiac death, and nonfatal myocardial infarction among women, and with increased risk of sudden and non-sudden cardiac death among ARIC men^25^. Similarly, Elfassy and colleagues observed that income volatility and more than 25% income drop were independently associated with increased risk of incident CVD and mortality among Coronary Artery Risk Development in Young Adults (CARDIA) participants^26^. Faselis et al. studied Cardiovascular Health Study (CHS) participants recruited in 1989–1993 and reported that low income (< $16,000) was associated with 16% higher CVD risk and 19% higher mortality risk during follow-up^27^. Our study builds upon existing data by showing that these income related disparities have persisted well into the twenty-first century.

Our study has several important implications. The findings of our study highlight greater prevalence of CVD risk factors, CVD, and mortality among lower income households. Clinicians, health systems, payors, policymakers, and other relevant stakeholders should devise targeted interventions to achieve equity in cardiovascular healthcare and outcomes across the spectrum of income-strata. Essential strategies include improving healthcare access, promoting health education, improving housing and food quality, alleviating poverty, and other widespread public health and policy efforts for integrating social determinants of health (SDOH) into clinical care to help clinicians provide targeted care to marginalized populations. Further investigation is critical to determine the factors responsible for deleterious effects of family income on CVD prevalence. Research with robust study designs should focus on identifying the risk factors, causative mechanisms and systematic differences resulting in the disparate healthcare outcomes among different income strata.

### Strengths and limitations

A major strength of this study is the well-established nationwide cohort in the US. The sample size is large, diverse, and nationally representative which supports generalization of our findings. However, the findings of our study should be interpreted in the context of its limitations. Our report focuses on income disparity measured using the well-validated PIR, but PIR alone does not capture the total impact of SES on CVD and outcomes. However, we noted that the association of PIR with CVD remained significant after controlling for four other SES markers—educational attainment, marital status, citizenship status, and health insurance. The possibility of residual confounding cannot be excluded given the observational nature of this study. NHANES is a series of cross-sectional studies, participants were not followed longitudinally, and thus we were not able to capture non-fatal incident CVD events, only mortality events. Lastly, we cannot establish causality or direction of the association i.e. we cannot entirely exclude low family income due to prevalence of CVD.

## Conclusions

Our study demonstrates largely dose-dependent association of PIR with hypertension, diabetes, CHF, CAD, stroke, all-cause mortality, and cardiac mortality. Public and policy health efforts should be directed to alleviate disparities and improve cardiovascular outcomes in vulnerable subgroups of the population.

## Supplementary Information

Supplementary Information.
