# Supplementary material for: Family income and cardiovascular disease risk in American adults
Source: Sci Rep. 2023 Jan 6;13:279. doi: 10.1038/s41598-023-27474-x (PMC9822929; doi:10.1038/s41598-023-27474-x)
Supplement: Supplementary file 1 — Supplementary Information. [file 41598_2023_27474_MOESM1_ESM.docx]

**Supplemental Data:**

**Supplementary Table 1**: Prevalence of cardiovascular comorbidities over time stratified by PIR

|  | **Unadjusted; Weighted %** | | | | | | | | | **Adjusted*** | | | | | | | |
| --- | --- | --- | --- | --- | --- | --- | --- | --- | --- | --- | --- | --- | --- | --- | --- | --- | --- |
|  | **2005-2006** | **2007-2008** | **2009-2010** | **2011-2012** | **2013-2014** | **2015-2016** | **2017-2018** | **Total** | **P-Trend** | **2005-2006** | **2007-2008** | **2009-2010** | **2011-2012** | **2013-2014** | **2015-2016** | **2017-2018** | **P-Trend** |
| **Heart Failure** PIR< 1 | 3.45 | 2.89 | 2.33 | 3.93 | 3.37 | 4.6 | 2.16 | 3.29 | 0.688 | 1.40 | 1.29 | 1.11 | 1.81 | 1.48 | 1.90 | 0.94 | 0.789 |
| PIR 1- 1.9 | 3.91 | 3.79 | 2.75 | 4.03 | 4.79 | 4.24 | 4.05 | 3.96 | 0.357 | 0.82 | 0.87 | 0.68 | 1.00 | 1.16 | 1.06 | 1.06 | 0.077 |
| PIR 2-2.9 | 4.54 | 3.18 | 2.64 | 3.44 | 2.05 | 1.65 | 3.06 | 2.91 | 0.11 | 0.90 | 0.56 | 0.47 | 0.63 | 0.40 | 0.34 | 0.50 | 0.141 |
| PIR 3-3.9 | 2.27 | 1.76 | 2.31 | 1.53 | 2.61 | 2.31 | 1.46 | 2.06 | 0.712 | 0.44 | 0.36 | 0.43 | 0.29 | 0.42 | 0.39 | 0.24 | 0.392 |
| PIR 4-4.9 | 1.12 | 0.92 | 0.82 | 2.95 | 2.15 | 1.44 | 1.47 | 1.55 | 0.341 | 0.23 | 0.19 | 0.12 | 0.45 | 0.34 | 0.22 | 0.20 | 0.838 |
| PIR>5 | 0.74 | 0.64 | 0.97 | 0.84 | 1.01 | 0.54 | 1.39 | 0.88 | 0.288 | 0.07 | 0.06 | 0.08 | 0.07 | 0.07 | 0.04 | 0.09 | 0.939 |
| **Stroke** PIR< 1 | 4.66 | 3.92 | 3.48 | 4.31 | 4.01 | 3.97 | 4.67 | 4.12 | 0.811 | 2.33 | 2.10 | 2.00 | 2.36 | 2.10 | 1.92 | 2.44 | 0.996 |
| PIR 1- 1.9 | 3.87 | 4.88 | 3.71 | 4.35 | 4.29 | 4.66 | 4.44 | 4.32 | 0.596 | 0.91 | 1.29 | 1.05 | 1.24 | 1.14 | 1.34 | 1.34 | 0.122 |
| PIR 2-2.9 | 4.71 | 4.67 | 2.55 | 3.02 | 4.16 | 2.25 | 3.87 | 3.58 | 0.201 | 1.31 | 1.23 | 0.64 | 0.79 | 1.23 | 0.67 | 0.95 | 0.211 |
| PIR 3-3.9 | 2.37 | 1.8 | 2.63 | 2.36 | 2.22 | 3.53 | 1.88 | 2.39 | 0.808 | 0.66 | 0.51 | 0.71 | 0.62 | 0.49 | 0.85 | 0.40 | 0.649 |
| PIR 4-4.9 | 1.36 | 1.39 | 1.85 | 1.5 | 1.67 | 1.68 | 2.43 | 1.71 | 0.243 | 0.13 | 0.15 | 0.13 | 0.10 | 0.12 | 0.12 | 0.15 | 0.924 |
| PIR>5 | 1.01 | 1.83 | 1.8 | 1.51 | 0.86 | 1.18 | 2.32 | 1.5 | 0.418 | 0.15 | 0.28 | 0.24 | 0.18 | 0.10 | 0.14 | 0.23 | 0.633 |
| **CAD** PIR< 1 | 7.09 | 7.37 | 6.06 | 6.14 | 7.3 | 7.32 | 6.33 | 6.78 | 0.907 | 3.59 | 4.00 | 3.47 | 3.24 | 3.90 | 3.43 | 3.17 | 0.495 |
| PIR 1- 1.9 | 8.81 | 7.5 | 7.12 | 6.31 | 9.3 | 8.78 | 7.08 | 7.85 | 0.978 | 2.10 | 1.86 | 1.97 | 1.64 | 2.47 | 2.41 | 1.97 | 0.431 |
| PIR 2-2.9 | 8.22 | 5.57 | 4.8 | 8.47 | 5.65 | 5.32 | 8.79 | 6.67 | 0.806 | 2.18 | 1.28 | 1.11 | 2.14 | 1.50 | 1.54 | 2.05 | 0.72 |
| PIR 3-3.9 | 4.11 | 4.22 | 5.35 | 2.74 | 6.7 | 6.9 | 5.35 | 5.06 | 0.109 | 0.73 | 0.83 | 0.95 | 0.44 | 1.05 | 1.11 | 0.86 | 0.358 |
| PIR 4-4.9 | 4.56 | 2.92 | 3.76 | 5.32 | 4.7 | 4.92 | 3.47 | 4.22 | 0.813 | 0.61 | 0.36 | 0.32 | 0.50 | 0.47 | 0.47 | 0.27 | 0.374 |
| PIR>5 | 3.46 | 4.42 | 5.4 | 3.09 | 3.09 | 3.04 | 6.93 | 4.2 | 0.253 | 0.46 | 0.59 | 0.62 | 0.32 | 0.29 | 0.29 | 0.66 | 0.642 |
| **Hypertension** PIR< 1 | 44.08 | 45.18 | 45.37 | 47.1 | 48.31 | 50.46 | 51.29 | 47.53 | 0.016 | 35.76 | 38.27 | 39.66 | 40.04 | 39.77 | 39.09 | 41.59 | 0.274 |
| PIR 1- 1.9 | 53.01 | 51.66 | 51.88 | 52.03 | 56.44 | 52.08 | 52.76 | 52.88 | 0.67 | 36.82 | 35.34 | 36.60 | 36.28 | 41.56 | 37.43 | 38.32 | 0.311 |
| PIR 2-2.9 | 50.46 | 50.75 | 51.39 | 53.55 | 52.36 | 48.38 | 57.15 | 51.94 | 0.247 | 35.26 | 33.78 | 34.83 | 36.04 | 39.25 | 35.35 | 41.33 | 0.106 |
| PIR 3-3.9 | 51.42 | 47.93 | 50.98 | 48.86 | 46.53 | 56.62 | 54.92 | 51.03 | 0.282 | 37.78 | 33.62 | 36.19 | 34.04 | 28.12 | 40.07 | 38.77 | 0.862 |
| PIR 4-4.9 | 44.21 | 49.7 | 47.22 | 54.39 | 49.6 | 42.24 | 50.54 | 48.33 | 0.73 | 34.29 | 37.26 | 31.71 | 38.59 | 33.22 | 25.75 | 34.37 | 0.371 |
| PIR>5 | 49.76 | 48.9 | 43.72 | 50.67 | 47.91 | 50.86 | 52.01 | 49.18 | 0.254 | 37.24 | 35.27 | 27.99 | 34.93 | 32.07 | 34.60 | 34.23 | 0.65 |
| **Obesity** PIR< 1 | 35.46 | 35.23 | 37.52 | 36.57 | 39.78 | 42.85 | 44.86 | 38.93 | <0.001 | 31.68 | 32.23 | 33.97 | 33.84 | 36.02 | 39.45 | 42.05 | <0.001 |
| PIR 1- 1.9 | 38.53 | 35.23 | 37.92 | 41.56 | 41.98 | 41.32 | 42.95 | 40.03 | 0.004 | 36.17 | 33.26 | 35.78 | 40.14 | 39.95 | 39.66 | 41.34 | 0.002 |
| PIR 2-2.9 | 34.82 | 36.41 | 37.59 | 35.26 | 44.91 | 39.28 | 47.52 | 39.44 | <0.001 | 34.26 | 35.68 | 36.77 | 34.79 | 44.72 | 38.75 | 47.53 | <0.001 |
| PIR 3-3.9 | 36.89 | 32.12 | 39.59 | 35.32 | 38.65 | 44.01 | 41.41 | 38.34 | 0.045 | 35.65 | 30.22 | 38.57 | 34.21 | 37.18 | 42.70 | 40.74 | 0.029 |
| PIR 4-4.9 | 32.88 | 38.03 | 36.11 | 38.69 | 35.36 | 44.01 | 41.15 | 38.14 | 0.075 | 31.14 | 36.24 | 33.80 | 35.97 | 33.19 | 41.55 | 38.68 | 0.123 |
| PIR>5 | 30.12 | 28.85 | 31.44 | 26.91 | 30.37 | 35.04 | 41.62 | 32.22 | 0.001 | 29.01 | 27.84 | 30.27 | 25.65 | 29.43 | 33.97 | 40.47 | 0.001 |
| **Dyslipidemia** PIR< 1 | 29.84 | 30.05 | 27.79 | 34.25 | 32.92 | 34.16 | 32.19 | 31.84 | 0.05 | 22.50 | 23.26 | 21.49 | 27.84 | 25.21 | 23.87 | 23.15 | 0.52 |
| PIR 1- 1.9 | 36.39 | 34.75 | 36.16 | 37.85 | 41.62 | 39.8 | 37.44 | 37.82 | 0.049 | 21.89 | 20.87 | 23.39 | 24.67 | 27.68 | 27.03 | 24.98 | 0.001 |
| PIR 2-2.9 | 41.2 | 39.1 | 39.02 | 43.91 | 40.53 | 40.59 | 41.53 | 40.83 | 0.723 | 28.11 | 24.92 | 24.93 | 28.86 | 28.51 | 29.49 | 26.74 | 0.429 |
| PIR 3-3.9 | 33.57 | 40.22 | 33.42 | 39.5 | 47.93 | 41.96 | 43.2 | 39.84 | 0.001 | 22.14 | 28.62 | 21.68 | 26.97 | 32.70 | 27.39 | 28.39 | 0.02 |
| PIR 4-4.9 | 37.54 | 38.91 | 35.49 | 43.59 | 43.43 | 44.05 | 37.83 | 40.07 | 0.348 | 26.32 | 25.63 | 19.56 | 26.60 | 26.85 | 26.72 | 20.93 | 0.566 |
| PIR>5 | 41.68 | 41.09 | 40.08 | 48.02 | 45.79 | 45.54 | 48.14 | 44.42 | 0.014 | 29.74 | 28.39 | 25.88 | 33.48 | 30.90 | 30.44 | 31.95 | 0.205 |
| **Diabetes** PIR< 1 | 12.25 | 12.17 | 10.85 | 14.54 | 14.04 | 18.33 | 14.78 | 13.97 | 0.002 | 6.20 | 6.66 | 5.86 | 8.18 | 7.37 | 8.74 | 7.52 | 0.031 |
| PIR 1- 1.9 | 13.54 | 14.86 | 14.4 | 15.55 | 13.93 | 14.99 | 15.95 | 14.76 | 0.274 | 5.59 | 6.41 | 6.37 | 7.05 | 5.97 | 6.57 | 7.13 | 0.126 |
| PIR 2-2.9 | 10.07 | 12.23 | 13.18 | 12.42 | 13.18 | 13.53 | 17.28 | 13.15 | 0.012 | 4.57 | 5.35 | 5.99 | 5.44 | 6.39 | 6.72 | 7.70 | 0.01 |
| PIR 3-3.9 | 8.03 | 9.15 | 10.98 | 9.6 | 13.45 | 16.75 | 11.19 | 11.2 | 0.007 | 4.08 | 4.75 | 5.68 | 4.79 | 6.18 | 8.49 | 5.03 | 0.051 |
| PIR 4-4.9 | 5.37 | 11.7 | 9.47 | 13.54 | 14.73 | 11.16 | 11.94 | 11.1 | 0.02 | 2.80 | 6.21 | 4.01 | 6.01 | 6.66 | 4.74 | 4.79 | 0.353 |
| PIR>5 | 7.13 | 7.72 | 8.12 | 6.08 | 8.91 | 9.39 | 14.7 | 8.96 | <0.001 | 3.88 | 4.11 | 4.04 | 2.85 | 4.25 | 4.46 | 6.89 | 0.001 |

* Models are adjusted for age, race and sex

**Supplementary Table 2:** Odds ratios (95% confidence intervals) for the prevalence of cardiovascular comorbidities with PIR as a continuous variable

|  | **Unadjusted** | **P value** | **Adjusted*** | **P value** |
| --- | --- | --- | --- | --- |
| **Variables** | OR (95% Confidence Interval) |  | OR (95% Confidence Interval) |  |
| CHF | 0.75 (0.71-0.79) | <0.001 | 0.71 (0.66-0.75) | <0.001 |
| CAD | 0.86 (0.83-0.89) | <0.001 | 0.81 (0.77-0.84) | <0.001 |
| Stroke | 0.78 (0.75-0.82) | <0.001 | 0.75 (0.71-0.80) | <0.001 |
| Dyslipidemia | 1.09 (1.07-1.11) | <0.001 | 1.05 (1.03-1.07) | <0.001 |
| Diabetes | 0.89 (0.87-0.92) | <0.001 | 0.90 (0.88-0.93) | <0.001 |
| Hypertension | 0.99 (0.97-1.01) | 0.379 | 0.92 (0.90-0.94) | <0.001 |
| Obesity | 0.94 (0.92-0.96) | <0.001 | 0.96 (0.94-0.98) | <0.001 |

* Models are adjusted for age, race/ethnicity and sex

Key: PIR: Family poverty to income ratio; CAD: Coronary artery disease; CHF: Congestive heart failure

**Supplementary Table 3:** Number of all cause deaths, cardiac deaths and follow-up time stratified by PIR Categories

| **All-cause mortality** | |  |  |  |  |  |  |  |
| --- | --- | --- | --- | --- | --- | --- | --- | --- |
| **Variables** | **PIR < 1** | **PIR 1- 1.9** | **PIR 2-2.9** | **PIR 3-3.9** | **PIR 4-4.9** | **PIR >=5** | **Total** | **P Value** |
| Weighted n | 30104438 | 42289598 | 30892173 | 27686092 | 20850477 | 52745517 | 204568295 |  |
| Unweighted n | 5,679 | 6,917 | 3,834 | 2,969 | 2,032 | 4,606 | 26,037 |  |
| Assumed alive, n (weighted %) | 5181(92.96) | 6131(90.78) | 3428(92.34) | 2759(95.12) | 1920(96.14) | 4420(97.34) | 23839(94.16) | <0.001 |
| All-cause mortality, n (weighted %) | 498(7.04) | 786(9.22) | 406(7.66) | 210(4.88) | 112(3.86) | 186(2.66) | 2198(5.84) |  |
| Mean (Maximum) follow up time | 5.4(11) | 5.6(11) | 5.8(11) | 5.9(11) | 6(11) | 5.9(11) | 5.7(11) |  |
| **Cardiac Mortality** | |  |  |  |  |  |  |  |
| **Variables** | **PIR < 1** | **PIR 1- 1.9** | **PIR 2-2.9** | **PIR 3-3.9** | **PIR 4-4.9** | **PIR >=5** | **Total** | **P Value** |
| Weighted n | 28341783 | 39047273 | 28893645 | 26477078 | 20169893 | 51509905 | 194439577 |  |
| Unweighted n | 5,273 | 6,272 | 3,497 | 2,786 | 1,937 | 4,446 | 24,211 |  |
| Assumed alive, n (weighted %) | 5181(98.74) | 6131(98) | 3428(98.73) | 2759(99.46) | 1920(99.39) | 4420(99.68) | 23839(99.07) | <0.001 |
| Cardiac mortality, n (weighted %) | 92(1.26) | 141(1.68) | 69(1.27) | 27(0.54) | 17(0.61) | 26(0.32) | 372(0.93) |  |
| Mean (Maximum) follow up time | 5.5(11) | 5.7(11) | 6(11) | 6(11) | 6.1(11) | 6(11) | 5.8(11) |  |

Key: PIR: Family poverty to income ratio

**Supplementary Figure 1:** Kaplan-Meier curves for all-cause mortality stratified by PIR Categories (A) unadjusted, (B) adjusted for age, sex and race/ethnicity
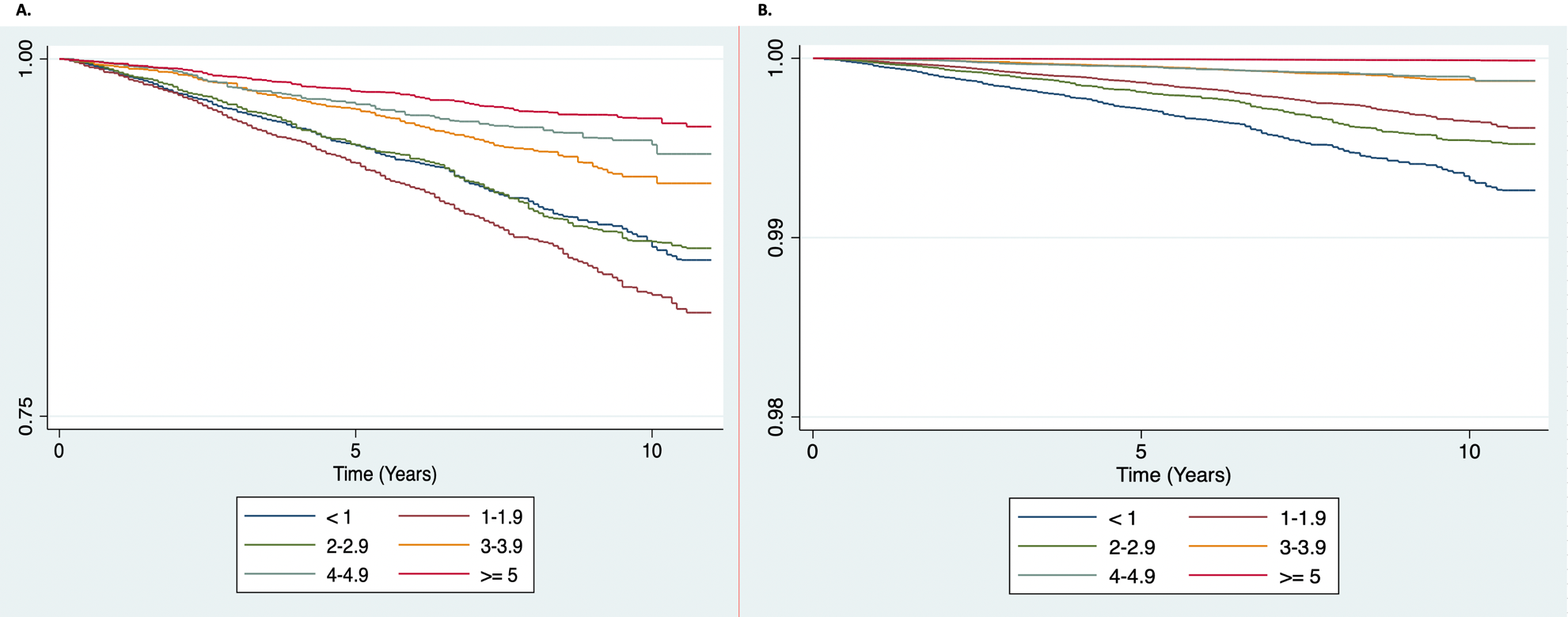


Key: PIR: Family poverty to income ratio

**Supplementary Figure 2:** Kaplan-Meier curves for cardiac mortality stratified by PIR Categories (A) unadjusted, (B) adjusted for age, sex and race/ethnicity


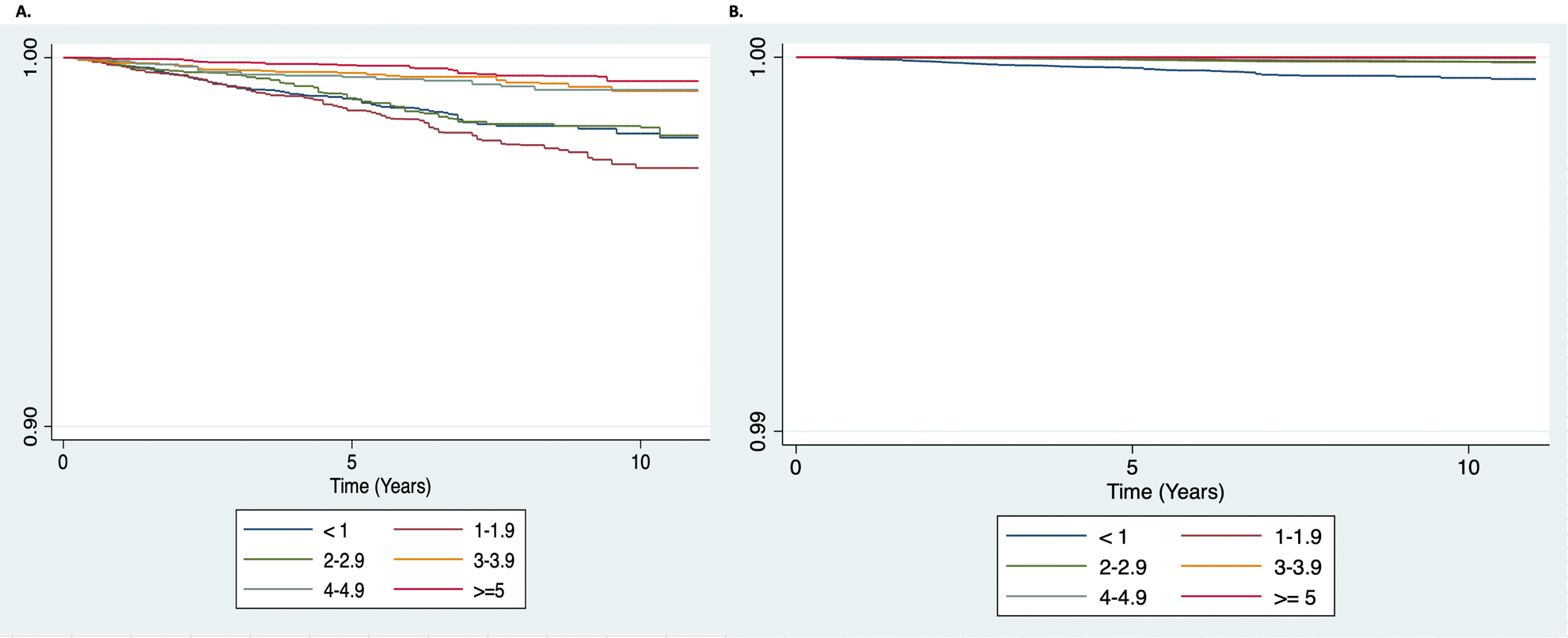


Key: PIR: Family poverty to income ratio
